# In This Issue

**DOI:** 10.1002/cfg.417

**Published:** 2004-07


**Comparative and Functional Genomics**
*Comp Funct Genom* 2004; **5**: 381.
Published online in Wiley InterScience (www.interscience.wiley.com). **DOI:** 10.1002/cfg.417

### In this issue

### A critical and integrated view of the yeast interactome

Michael Cornell and colleagues have used the Genome Information Management System (GIMS) to integrate interactome data sets with transcriptome and protein annotation data. They have analysed the overlap between datasets and estimated the proportion of false positives generated by global interaction analysis approaches.

### Gonad transcriptomes in zebrafish

In their paper, Yang Li *et al.* describe the construction of adult zebrafish testis and ovary cDNA libraries, and the subsequent production of 'Gonad UniClone' macroarrays containing unique cDNA clones. These arrays were used to profile mature adult testis and ovary samples to identify genes with organ-specific expression patterns.

### Profiling the heat shock response of a yeast *PDE2* deletant

Loss of the *PDE2* gene in *Saccharomyces cerevisiae* results in a constitutively active cAMP-dependent pathway and reduced ability to tolerate a range of stresses. Dawn Jones and colleagues have used microarrays to profile the heat shock response of such a *PDE2* deletant. The genes identified in this study could play a role in the drastic reduction of viability seen in this mutant after heat shock.

### Choosing normalization methods and test statistics for microarrays

Yang Xie *et al.* describe a practical approach to addressing key questions in array data analysis relating to normalization and test statistics. They recommend using an empirical Bayes method (the *B* statistic) for comparative inference, showing it to perform better than other test statistics on two types of array data.

### Conference calendar

A listing of genomics-related conferences planned for November 2004–January 2005.

Copyright © 2004 John Wiley & Sons, Ltd.

